# Spasmodic Dyspnœa Cured by Purgatives

**Published:** 1831-01-01

**Authors:** 


### SURREY DISPENSARY.

### SPASMODIC DYSPNŒA CURED BY PURGATIVES.

AN unmarried female, aged 22 years, was admitted on the 22d of August, under Dr. Whiting. Eight years previously, during convalescence from fever, she became subject to hysterical fits, recurring regularly at stated periods, up to the time of admission. Four years previous to the said admission, she was attacked with the disease for which she now sought relief. She had laboured under obstinate constipation. Being under a paroxysm of the disease at the time of admission, the following phenomena were noted by the reporter, Mr. Grey:—

"All the muscles employed in breathing are thrown into violent spasmodic action; the shoulders are forcibly elevated at each inspiration, and cannot be restrained by the strongest pressure; the nostrils are dilated, the countenance flushed and anxious, and she has exactly the appearance of one who is out of breath from the most violent exertion. The respirations are about 60 in the minute, the heart beats forcibly, and the pulse is very much accelerated. The fit has generally continued two or three days, sometimes a week, and once even as long as a month. She states that, when it is very severe, she is obliged to support herself in a semi-erect posture by bolsters, sometimes passing two or three days together without sleep; at length, however, she becomes excessively drowsy, falls asleep, and the respiration returns to its natural condition. The paroxysms have usually recurred after short intervals, but even when free from them, the breathing is quicker than natural. She complains during the paroxysms of a distressing sense of tightness across the sternum. The true hysteric fits, above alluded to, frequently occur both during the attacks of dyspnœa and in the intervals. She states that she is enabled to predict the return of dyspnœa by a sense of sickness, and by pain at the lower part of the sternum, with a voracious and depraved appetite. There is, both during the paroxysms and in the intervals, a constant pain under the left mamma, not increased by pressure. She complains also of a dull aching pain across the abdomen, especially just above the iliac fossæ. The bowels are obstinately confined, her motions very infrequent, hard, and passed with much difficulty; she has a voracious appetite; the tongue is clean; the pulse natural in the absence of the fits; the catamenia have been regular from the commencement of her disease, although there is always considerable pain at the menstrual periods.

She had been frequently under medical treatment for this complaint before her admission into the Surrey Dispenpensary, and had taken almost all the powerful antispasmodic medicines in the Pharmacopœia without any effect."*

The patient was put on a plan of purgation—chiefly powdered aloes and ipecacuan at night, with castor oil in the morning—and this course was pursued, with some interruptions, and a few bleedings, general and topical, from the 22d August to the 21st December, during which she had repeated attacks of the complaint above described. On the last-mentioned date she was discharged cured, and for a period of nine months afterwards, she was able to officiate as cook in a private family, with only a few slight returns of the dyspnœa, which always gave way to purgatives.

With the disposition to give all due credit to the Hamiltonian discipline which the above patient underwent during a period of 123 days, it is just possible that other plans of treatment might have been equally successful in that, or even in a shorter space of time. The usual dose of purgative medicine was seven grains of powdered aloes and two grains of ipecacuan every night, with three drachms of castor oil every morning. These medicines generally acted powerfully, and sometimes distressingly so. Thus, as an example, "15th September, since the last report (on the 11th) the evacuations have been very frequent, watery, and unmixed with scybalæ. *The fit has become more severe.*" It is to be regretted that no examination of the chest appears to have been made, during all this long course of drastic purgation. Did the medical superintendants determine at once that the dyspnœa and hysteria were symptomatic of constipation, and that it was unnecessary to examine into any other phenomena, auscultic or semiotic? We have seen more distressing and anomalous symptoms give way to *moderate* evacuations from the bowels—regulated diet—and powerful counter-irritation to various parts of the surface.—*Verbum sapientibus.*

---

* Med. Gazette, No. 148.

